# The complete chloroplast genomes of two Mexican plants of the annual herb *Datura stramonium* (Solanaceae)

**DOI:** 10.1080/23802359.2020.1789516

**Published:** 2020-07-15

**Authors:** I. M. De-la-Cruz, J. Núñez-Farfán

**Affiliations:** Department of Evolutionary Ecology, Institute of Ecology, National Autonomous University of Mexico, Mexico City, Mexico

**Keywords:** Chloroplast assembly, *Datura stramonium*, Organelle, Solanaceae

## Abstract

The annual herb, *Datura stramonium*, is a member of the Solanaceae family. In this study, we report the chloroplast genomes of two Mexican plants of *D. stramonium*. Both chloroplast genomes of *D. stramonium* (GenBank accessions: MT610896 and MT610897) were assembled as a circular molecule. The genome size of both plants was similar (155,884 bp). The overall GC content was 38.59% for both genomes. Both chloroplast genomes contained 85 protein-coding sequences (CDS), 131 genes, 8 rRNA genes, and 38 tRNA genes. Thirty-nine microsatellites (SSRs) and 42 long tandem repeats were also identified for both genomes. The phylogenetic relationship between *D. stramonium* and related Solanaceae species revealed four main groups; *Nicotiana*, *Datura*, *Capsicum*, and *Solanum* clades. This species tree is consistent with other Solanaceae species trees already published.

## Chloroplast genome announcement

The annual herb, *Datura stramonium*, is a member of the Solanaceae family (Castillo et al. 2019). This herb produces the highest concentration of tropane alkaloids within this family (Castillo et al. 2013; Kohnen-Johannsen and Kayser 2019; De-la-Cruz et al. 2020). *Datura stramonium*, although native to North America, has expanded its distribution, owing to humans, worldwide except to polar and subpolar climate zones (Weaver and Warwick 1984). This species occurs, distinctively, in human-disturbed habitats (Weaver and Warwick 1984; Núñez-Farfán and Dirzo 1984). Recently, the first draft nuclear genomes of two Mexican plants of *D. stramonium* have been released (DDBJ/ENA/GenBank BioProject: PRJNA622882; De-la-Cruz et al. *in prep*). One nuclear genome corresponds to a plant collected in Ticumán, State of Morelos, Mexico (GenBank accession JAAWWX000000000), and the other nuclear genome corresponds to a plant collected in Teotihuacán, State of Mexico, Mexico (GenBank accession JAAWWY000000000). DNA of both genomes has been stored at the Laboratory of Ecological Genetics and Evolution of the Institute of Ecology at the National Autonomous University of Mexico. Specimens are stored in the Botanic Garden of the Institute of Biology at the National Autonomous University of Mexico.

In this study, we report the chloroplast genomes of these two plants of *D. stramonium.* To this end, gDNA was extracted from fresh leaves with a modified CTAB mini-prep protocol (Doyle and Doyle 1987). A total of 200 ng of gDNA were used to construct paired-end (PE) libraries for Illumina HiSeq 4000 sequencing. The sequencing and library preparation were carried out in the QB3 Functional Genomics and Vincent J. Coates Sequencing Laboratories at the University of California, Berkeley. 323M PE raw sequences (2 × 150b; ∼300 bp insert size) were obtained from Illumina sequencing; corresponding to 112 Gb and an average 30.85-fold genome coverage for the Ticumán individual, while 318M PE sequences corresponding to 110 Gb and 30.29-fold genome coverage were generated for the Teotihuacán individual. Trimming of Illumina sequences was carried out using a Phred quality score > 30 in TRIMMOMATIC v0.32 (Bolger et al. 2014). Then, NOVOPlasty v3.8.2 (Dierckxsens et al. 2017) was used to assembly the chloroplast genomes. We used the subsample option in NOVOPlasty to limit the RAM memory that the program can allocate. Thus, the program takes a fraction of the millions of PE raw sequences based on the RAM memory allowed by the user (i.e. 40 Gb). The program subsampled 15.10% of the PE sequences in both datasets (2,168,304 sequences for the Teotihuacán plant and 1,545,934 for the Ticumán plant).

NOVOPlasty has to be initiated using a seed (Dierckxsens et al. 2017). As we have already assembled the nuclear genomes of both plants, we used DIAMOND blast (*E*-value < 1e − 5) (Buchfink et al. 2015) to align the reference chloroplast genome of *D. stramonium* (Yang et al. 2014; GenBank accession NC_018117) to the two *Datura* nuclear genome assemblies. We retrieved the contigs from the *Datura* assemblies that had a matching value of 100% with the reference chloroplast. These contigs were used as a seed in NOVOPlasty to reconstruct completely the chloroplasts of our *D. stramonium* plants. Structural and functional annotation of both chloroplast genomes was carried out using the program GeSeq with default parameters (Tillich et al. 2017). tRNAscan-SE v2.0.5 was used to find and annotate tRNA genes. Both chloroplast genomes of *D. stramonium* (GenBank accession: MT610896 and MT610897, Ticumán and Teotihuacán, respectively) were assembled as a circular molecule. Both genomes had the same genome size (155,884 bp). The overall GC content was 38.59% for both genomes. Both chloroplast genomes contained 85 protein-coding sequences (CDS), 131 genes, 8 rRNA genes, and 38 tRNA genes. Thirty-nine microsatellites (SSRs) and 42 long tandem repeats were also identified for both genomes.

**Figure 1.**
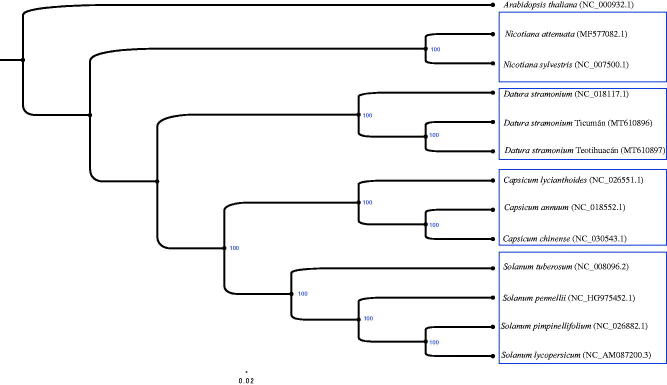
ML phylogenetic tree of *D. stramonium* with 13 species was constructed by chloroplast sequence. Numbers in the nodes are bootstrap values from 1000 replicates. *Arabidopsis thaliana* was set as outgroup. NCBI accession of each chloroplast genome is indicated within the parenthesis.

To understand the phylogenetic relationship between *D. stramonium* and related species, the complete chloroplast of 13 species was aligned using the program MAFFT v7 (Katoh et al. 2002). The chloroplast of *A. thaliana* was used as outgroup. The evolutionary history was inferred with the program RAxML-NG (Kozlov et al. 2019) (options; random starting tree, general time-reversible model, maximum-likelihood estimate of substitution rates and nucleotide frequencies, discrete GAMMA model of rate heterogeneity with four categories and 1000 bootstrap replicates). The phylogenetic tree was divided into four groups: *Nicotiana*, *Datura*, *Capsicum*, and *Solanum* clades (Figure 1). *Arabidopsis thaliana* was selected as outgroup. This species tree is consistent with other Solanaceae species trees already published (Olmstead et al. 2008).

## Data Availability

The workflow, commands, chloroplast genome annotation and the complete chloroplast genomes in Fasta file can be consulted in the GitHub repository; https://github.com/icruz1989/ChloroplastofDaturastramonium. The complete chloroplast genomes used for the phylogenetic analysis were retrieved from NCBI; *Arabidopsis thaliana* (NC_000932.1), *Nicotiana attenuata* (MF577082.1), *Nicotiana sylvestris* (NC_007500.1), *Datura stramonium* (NC_018117.1), *Datura stramonium* Ticumán (MT610896), *Datura stramonium* Teotihuacán (MT610897), *Capsicum lycianthoides* (NC_026551.1), *Capsicum annuum* (NC_018552.1), *Capsicum chinense* (NC_030543.1), *Solanum tuberosum* (NC_008096.2), *Solanum pennellii* (NC_HG975452.1), *Solanum pimpinellifolium* (NC_026882.1), *Solanum lycopersicum* (NC_AM087200.3).
